# Resistance of Wild *Solanum* Accessions to Aphids and Other Potato Pests in Quebec Field Conditions

**DOI:** 10.1673/031.010.14121

**Published:** 2010-09-24

**Authors:** B. Fréchette, M. Bejan, É. Lucas, P. Giordanengo, C. Vincent

**Affiliations:** ^1^ Université du Québec à Montréal, Groupe de Recherche en Écologie Comportementale et Animale, Département des sciences biologiques, C.P. 8888, Succ. Centre-Ville, Montréal, Qc, Canada, H3C 3P8; ^2^ Centre de Recherche et de DeÏveloppement en Horticulture, Agriculture et Agro-alimentaire Canada, 430 Boul. Gouin, Saint-Jean-sur-Richelieu, Qc, Canada, J3B 3E6; ^3^ Université de Picardie Jules Verne, Biologie des Plantes et Contrôle des Insectes Ravageurs, 33 rue St Leu, 80039 Amiens cedex I, France

**Keywords:** Aphididae, Coleoptera, Plant resistance to herbivores, *Empoasca fabae*, *Epitrix cucumeris*, *Leptinotarsa decemlineata*, *Macrosiphum euphorbiae*, *Myzus euphorbiae*, *Solanum polyadenium*, *Solanum tarijense*

## Abstract

Two experiments were done to determine the susceptibility of six wild potato accessions to the aphids *Macrosiphum euphorbiae* (Thomas) (Hemiptera: Aphididae) and *Myzus persicae* (Sulzer). Densities of aphid colonies were compared between caged *Solanum pinnatisectum* Dunal (Solanales: Solanaceae), *S. polyadenium* Greenmam, *S. tarijense* Hawkes, *S. infundibuliforme* Philippi, *S. oplocense* Hawkes, and *S. stoloniferum* Schlechted and Bouché, and the commercially cultivated potato plant *S. tuberosum* L. cv. *Désirée*. Moreover the susceptibility of *S. polyadenium* and *S. tarijense* to the Colorado potato beetle *Leptinotarsa decemlineata* (Say) (Coleoptera: Chrlysomelidae), the potato flea beetle *Epitrix cucumeris* (Harris), and the potato leafhopper *Empoasca fabae* (Harris) (Hemiptera: Cicadellidae) was compared to that of *S. tuberosum* cv. *Désirée* in the field. Results indicated that *S. polyadenium* and *S. tarijense* were more resistant to *M. persicae* than *S. pinnatisectum* and the commercially cultivated *S. tuberosum* cv. *Désirée. Solanum polyadenium* was more resistant to aphids than *S. tarijense* in 2004, but not in 2005. Moreover, *S. polyadenium* and *S. tarijense* were more resistant than *S. tuberosum* cv. *Désirée* to *L. decemlineata, E. cucumeris* and *E. fabae*.

## Introduction

Worldwide potato production is hindered by a complex of insect pests. In Quebec, Canada, the Colorado potato beetle, *Leptinotarsa decemlineata* (Say) (Coleoptera: Chrysomelidae), the green peach aphid, *Myzus persicae* (Sulzer) (Hemiptera: Aphididae), and the potato aphid, *Macrosiphum euphorbiae* (Thomas), constitute the major pests in potato fields (Pelletier and Michaud 1995). While *L. decemlineata* adults and larvae are voracious defoliators of potato leaves, *M. persicae* and *M. euphorbiae* are important vectors of the two most damaging potato viruses (i.e. the potato leaf roll virus and the potato virus Y) (Radcliffe 1982; Boiteau et al. 1988). Pests which are normally of secondary importance such as the potato flea beetle, *Epitrix cucumeris* (Harris) (Coleoptera: Chrysomelidae), the potato leafhopper, *Empoasca fabae* (Harris) (Hemiptera: Cicadellidae), and the Buckthorn aphid, *Aphis nasturtii* (Hemiptera: Aphididae) may also occasionally cause serious damages and yield loss (Radcliffe 1982; Pelletier and Michaud 1995; Kaplan et al. 2008).

Development of insecticide resistance (Boiteau et al. 1987; van Toor et al. 2008) and public awareness of possible health problems associated with pesticides (Rimal et al. 2001) increased the interest for the development of alternative and sustainable control strategies for potato pests. One strategy that has received much attention is the development of insect-resistant potato cultivars (Flanders et al. 1992; Pelletier and Michaud 1995). In addition to the development of genetically modified plants, researchers have focused mainly on hybridization programs between commercially cultivated potato cultivars and related wild *Solanum* species resistant to several potato pests (Flanders et al. 1992) and diseases (Chen et al. 2003).

Laboratory and field experiments have identified many *Solanum* accessions as resistant to either one or a few potato pests (Flanders et al. 1992; LeRoux et al. 2007). Resistance mechanisms have been classified as either antibiosis (changes in insect biology and demographic parameters) or antixenosis (changes in insect behavior leading to low or non acceptance of the host plant) (LeRoux et al. 2008). In *Solanum* spp., antibiosis relies mainly on glycoalkaloids present in leaves (Stürckow and Löw 1961; Gregory et al. 1981; Güntner et al. 1997, 2000; Lachman et al. 2001; Lorenzen et al. 2001). For example, some species such as *Solanum pinnatisectum* Dunal (Solanales: Solanaceae) and *S. polyadenium* Greenmam have a high level of α-tomatine (Gregory et al. 1981; Sinden et al. 1991; Deahlt et al. 1993), which is known to hinder *L. decemlineata* growth (Kowalski et al. 2000) and lower *M. euphorbiae* reproductive rate (Güntner et al. 1997). High levels of α-chaconine and α-solanine also have negative impacts on *M. persicae* adults, lowering feeding and fecundity, and increasing mortality (Fragoyiannis et al. 1998). Antixenosis-based resistance may be conferred by glandular trichomes (Gibson 1971, 1976a; Tingey and Gibson 1978; Tingey and Laubengay er 1981; Neal et al. 1990; Pelletier and Smilowitz 1990; Yencho and Tingey 1994; Alvarez et al. 2006; Pelletier and Dutheil 2006). Glandular trichomes are known to alter the ability of many herbivores to colonize, forage, and survive on the plant. For example, glandular trichomes reduce the proportion of *L. decemlineata* larvae feeding on *S. polyadenium* and *S. berthaultii* leaves and increase larval mortality (Gibson 1976b; Neal et al. 1989).

Pest resistant plants may also have a negative impact on the third trophic level (Orr and Boethel 1986; Obrycki 1986). For example, glandular trichomes are known to hinder the foraging abilities of many insect predators (Arzet 1973; Elsey 1974; Belcher and Thurston 1982; Obrycki and Tauber 1984; Lucas et al. 2004; Gassmann and Hare 2005; Simmons and Gurr 2005) and parasitoids (Obrycki and Tauber 1984; Simmons and Gurr 2005). Moreover, chemicals responsible for antibiosis may also affect predators (Orr and Boethel 1986; Francis et al. 2001) and parasitoids (Ashouri et al 2001; Azzouz et al. 2005a, b).

On the other hand, certain plant characters conferring pest resistance are thought to have positive impacts on some natural enemies (Obrycki 1986). For example, the ladybird beetle *Coleomegilla maculata* (Griffin and Yeargan 2002a, b) lays eggs preferentially on plant species bearing glandular trichomes, and oviposition of the aphidophagous midge *Aphidoletes aphidimyza* is positively correlated with *S. tuberosum* trichome density (Lucas and Brodeur 1999). Obrycki and Tauber (1985) also observed positive relationship between ladybird eggs (unidentified species) and trichome abundance. For both ladybirds and *A. aphidimyza*, the preference for oviposition on trichome bearing plants may be associated with a lower predation risk of the most susceptible life stages on those plants (Lucas and Brodeur 1999; Griffin and Yeargan 2002a, b).

Studying the impact of *Solanum* spp. candidates on both pests and natural enemies is of importance for breeding programs, as natural enemies are known to contribute to aphid (Obrycki et al. 1983; Karley et al. 2003; Koss and Snyder 2005) and *L. decemlineata* (Hilbeck et al. 1997; Chang and Snyder 2004; Koss and Snyder 2005) biocontrol. The exclusion of natural enemies from resistant plants could provide an enemy-free space to herbivores adapted to resistant plants (Gassmann and Hare 2005).

Fields experiments are required in many different geographical areas since the expression of resistance characters may be lower in the field than in the laboratory (Tingey and Gibson 1978; Obrycki and Tauber 1984) and vary with environmental conditions (Gianfagna et al. 1992; Nihoul 1993). As such, resistant plants may have a different impact in the field than in controlled conditions, both on pests and on their natural enemies (Obrycki and Tauber 1984). Only a few field studies have tested the impact of resistant *Solanum* accessions on natural enemies.

To address that question, two experiments were done in Southern Quebec field conditions. The first compared the capacity of the aphids *M. persicae* and *M. euphorbiae* to thrive on six caged *Solanum* accessions and on caged potato plants. The second sampled and compared potato pest and natural enemy occurrence on two *Solanum* accessions and on potato plants.

## Materials and Methods

The experiments were performed from 2004 to 2007 on a commercial farm located at Saint-Paul d'Abbotsford (45.4127° N, 72.8598° W), Quebec, Canada. No insecticides, fungicides, or herbicides were applied to the experimental field.

Plants were previously grown in a greenhouse located at the Horticulture Research and Development Center, Agriculture and Agri-Food Canada, Saint-Jean-sur-Richelieu, Qc, Canada. Wild *Solanum* species seeds were obtained from the USDA Potato Introduction Project (Sturgeon Bay, Wisconsin, U.S.A.). The seeds were sown in early April in 25 × 50 cm plastic containers. Three weeks later, 30 seedlings of each variety were transplanted into pots (15 cm diameter). Potato plants *S. tuberosum* cv. *Désirée* were grown from tubers.

Aphids (*M. persicae* and *M. euphorbiae*) were reared on *S. tuberosum* cv. *Désirée*. One rearing unit was located at the Horticulture Research and Development Center, and the other was located at the University of Quebec in Montreal.

### Experiment 1 — Impact of six wild *Solanum* accessions on *Myzus persicae* and *Macrosiphum euphorbiae* colony development

The experiment was conducted in 2004 and 2005. The accessions used in 2004 were *S. pinnatisectum* PI 186553, *S. polyadenium* PI 230463, *S. tarijense* Hawkes PI 414150, *S. infundibuliforme* Philippi PI 458322, *S. oplocense* Hawkes PI 473368, and *S. stoloniferum stoloniferum* Schlechted and Bouché PI 201855. In 2005, only *S. pinnatisectum, S. polyadenium*, and *S. tarijense* were used due to time limitation. Both years, the commercially cultivated potato plant *Solanum tuberosum* cv. *Désirée* was used as a control.

Plantlets were transferred from the greenhouse to the field in early June both years, and were first placed in a shaded area to allow adaptation to field conditions. Three days later, nine plants of each accession and nine potato plants were transplanted in the field. Each plant species was transplanted in the field in three groups (a group consisted of 3 plantlets transplanted on a row). Both row spacing and planting distance on a row were set to 0.90 m. A muslin cage (Height: 1 m, Diameter: 0.60 m) was placed above each plant. The muslin at the base of each cage was buried in the soil at a depth of 20–30 cm and a lateral entry closed by a metal clip allowed access to the plant.

On 16 July 2004 and 13 July 2005 (i.e. four weeks after their transplantation in the field) each plant was infested with about 30 laboratory-reared apterous aphids (mixed instars). For each plant species, four plants were infested with *M. persicae*, and four plants were infested with *M. euphorbiae*. Infestation was done by placing 4 aphidinfested potato leaves on each plant.

Sampling started one week following aphid infestation. In 2004, all *Solanum* species were sampled weekly for four weeks. *Solanum pinnatisectum, S. polyadenium, S. tarijense*, and *S. tuberosum* cv. *Désirée* were sampled further for two weeks for a total of six weeks. In 2005, all plants were sampled weekly for six consecutive weeks. At each sampling date, the numbers of apterous and alate aphids were counted separately. All other insects found on the plants were removed.

### Experiment 2 — Impact of two wild *Solanum* accessions on aphids, *Leptinotarsa decemlineata, Epitrix cucumeris, Empoasca fabae*, and natural enemies

Experiment 2 was performed in 2007. It aimed at evaluating the natural occurrence of potato pests and natural enemies on *S. tarijense* PI 414150, *S. polyadenium* PI 230463, and *S. tuberosum* cv. *Désirée* in Southern Quebec field conditions. The accessions used were the same as in experiment 1.

Plantlets were transferred from the greenhouse to the field on 18 June 2007 and planted on 20 June 2007. The three accessions were planted in nine monospecific patches (three patches by plant species) laid out in a latin square. Each patch consisted of 7 × 7 plants. The distance between each patch was 1.80 m. The distance between plants corresponded to a typical plantation layout in southern Quebec, i.e. 0.30 m between plants on a row and 0.90 m between rows. Weeds were manually removed once or twice a week.

***Leptinotarsa decemlineata* survey.** On 29 June, 3 July, 6 July, 10 July, and 11 July 2007 all plants were inspected and all observed *L. decemlineata* eggs, larvae, and adults were removed. Eggs and adults were counted.

**Destructive sampling.** On 27 July 2007, 15 plants per patch were cut, individually enclosed in plastic bags, brought to the laboratory, and put in a freezer until inspection. Plants were carefully inspected and every insect collected was put in 70% alcohol for future identification. Specimens were identified in the laboratory using a dissecting microscope and a field guide.

### Statistical analysis

Data was analyzed using the statistical software JMP (SAS Institute 2001). Experiment 1: Data were rank-transformed and a MANOVA for repeated measures was applied followed by contrast analysis (between each pairs). In 2004 the analysis was done for four weeks in order to allow comparisons between the seven accessions tested, and then for six weeks for comparisons between *S. tuberosum* cv. *Désirée, S. pinnatisectum, S. polyadenium*, and *S. tarijense*. In 2005, the analysis was done for six weeks. Plants with missing data were excluded from the analysis (therefore, 1–4 plants/accession were compared for each analysis).

Experiment 2: For the *L. decemlineata* survey, the numbers of egg clutches and adults per row were compared (rows were used as experimental units). For the destructive sampling the numbers of individuals per plant were compared (individual plants were used as experimental units). This statistical methodology was used since the number of patches by accession was low (3 by accessions). Data were compared between accessions using Kruskal-Wallis tests and Tukey-type posthoc tests for non parametric data (Zar 1999).

## Results

### Experiment 1 — Impact of wild *Solanum* accessions on *Myzus persicae* and *Macrosiphum euphorbiae* colony development *Macrosiphum euphorbiae*.

**Figure 1.**
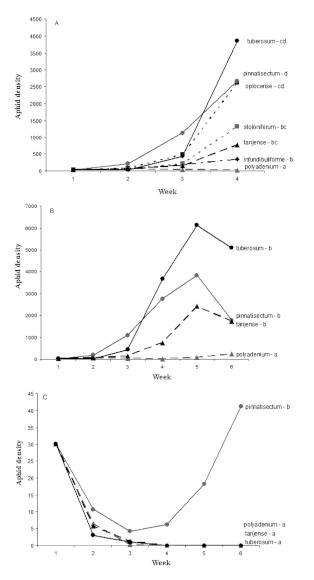
Mean number of *Macrosiphum euphorbiae* per plant (A) from week 1 to week 4 in 2004, (B) from week 1 to week 6 in 2004, and (C) from week 1 to week 6 in 2005 following an initial infestation of 30 aphids per plants in week 1. Different letters after the species names indicate significant difference (*P* < 0.05). High quality figures are available online.

In 2004, considering only the first four weeks, *M. euphorbiae* densities differed between the seven *Solanum* species (Figure 1a: F = 4.46; d.f. = 6, 18; P < 0.0001). *Solanum polyadenium* hosted the lowest *M. euphorbiae* density. Considering the whole experimental period, *M. euphorbiae* densities were significantly different between the four species present during the six sampling weeks (Figure 1b: F = 8.52; d.f. = 3, 8; P = 0.0003). Again, *S. polyadenium* had the lowest *M. euphorbiae* density.

In 2005, *M. euphorbiae* densities were significantly different between the four species (Figure 1c: F = 1.78; d.f. = 3, 12; P = 0.0053). *Solanum pinnatisectum* hosted significantly more aphids than *S. tuberosum* cv. *Désirée, S. tarijense*, and *S. polyadenium*, which were not significantly different from each other. Peak *M. euphorbiae* densities were much lower in 2005 than in 2004.

**Figure 2.**
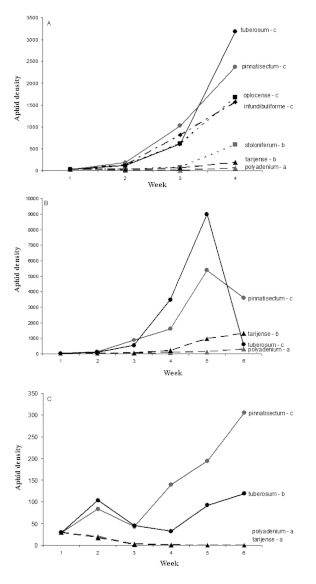
Mean number of *Myzus persicae* per plant (A) from week 1 to week 4 in 2004, (B) from week 1 to week 6 in 2004, and (C) from week 1 to week 6 in 2005 following an initial infestation of 30 aphids per plants in week 1. Different letters after the species names indicate significant difference (*P* < 0.05). High quality figures are available online.

***Myzus persicae*.** In 2004, considering only the first four weeks, there was a significant difference in the number of *M. persicae* between the seven *Solanum* species considered (Figure 2a: F = 10.23; d.f. = 6, 20; P < 0.0001). *Solanum polyadenium* had the lowest density, while *S. tuberosum* cv. *Désirée, S. pinnatisectum, S. oplocense*, and *S. infundibuliforme* had the highest densities. *Solanum stoloniferum* and *S. tarijense* hosted intermediate aphid densities. For the whole experimental period, *M. persicae* densities were significantly different between the four species present during the six sampling weeks (Figure 2b: F = 16.52; d.f. = 3, 7; P = 0.0001). *Solanum polyadenium* had the lowest density, while *S. tuberosum* cv. *Désirée* and *S. pinnatisectum* had the highest. *Solanum tarijense* hosted intermediate *M. euphorbiae* density.

In 2005, *M. persicae* densities were significantly different between the four species (Figure 2c: F = 4.59; d.f. = 3, 12; P < 0.0001). *Solanum polyadenium* and *S. tarijense* had the lowest densities, while *S. pinnatisectum* hosted the highest density. Peak *M. persicae* densities were much lower in 2005 than in 2004.

Except for the *M. euphorbiae* analysis of 2005 and for the six week analysis of *M. euphorbiae* in 2004 (P > 0.05), Wilk's Lamda test indicated P < 0.05 for “accession by time interaction”. However, Roy's Max Root test indicated P < 0.05 for all analysis.

**Experiment 2 — Impact of two wild *Solanum* accessions on aphids, *Leptinotarsa decemlineata, Epitrix cucumeris, Empoasca fabae*, and natural enemies *Leptinotarsa decemlineata* survey.** On most sampling dates, more egg clutches and adults were collected on *S. tuberosum* cv. *Désirée* than on *S. poyadenium* and *S. tarijense* (Table 1). There were no significant differences between *S. polyadenium* and *S. tarijense* regarding egg clutch and adult densities.

**Table 1.**
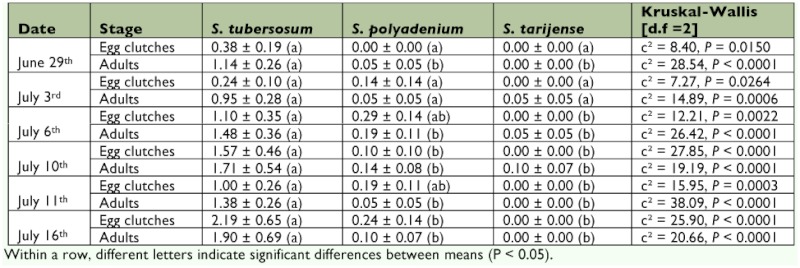
Mean (± SE) numbers of *Leptinotarsa decemlineata* egg clutches and adults per row per patch (i.e. 7 plants) on *Solanum tuberosum* cv. *Désirée*, *S. polyadenium*, and *S. tarijense* collected on 6 different dates in 2007.

**Destructive sampling.** Results of the destructive sampling are presented in Table 2. Although only a few *E. cucumeris* adults were observed, all were found on *S. tuberosum* cv. *Désirée* (Kruskal-Wallis: χ^2^ = 33.41, d.f. = 2, P < 0.0001). The low number of individuals collected (19) suggest however that this result should be interpreted cautiously.

A total of 156 leafhoppers (both nymphs and adults) were collected, and 155 of those leafhoppers were collected on *S. tuberosum* cv. *Desirée*, while only 1 occurred on *S. polyadenium* (Kruskal-Wallis: χ^2^ = 68.88, d.f. = 2, P < 0.0001). No leafhoppers were found on *S. tarijense*. Most leafhoppers were identified as *E. fabae* (94.2%), the remaining being too damaged to be formally identified.

During the destructive sampling, the distribution of the 1656 recovered *L. decemlineata* eggs significantly differed with 99.2% sampled on *S. tuberosum* cv. *Désirée*, 0.8% on *S. polyadenium*, and none on *S. tarijense* (Kruskal-Wallis: χ^2^ = 35.28, d.f. = 2, P < 0.0001). Similar results were obtained for *L. decemlineata* 1^st^ instars (228 individuals, 97.4% on *S. tuberosum*, 2.6% on *S. polyadenium*) and second instars (233 individuals, 99.1% on *S. tuberosum*, 0.4% on *S. tarijense*, and 0.4% on *S. polyadenium*) (Kruskal-Wallis: χ^2^_L1_ = 27.21, d.f. = 2, P < 0.0001; χ^2^_L2_ = 27.03, d.f. = 2, P < 0.0001). The higher proportion of third instar larvae on *S. tuberosum* cv. *Désirée* was not as pronounced than for the first and second instar larvae (28 individuals, 89.3% on *S. tuberosum* and 10.7% on *S. polyadenium*), and even though there was a global significant difference (Kruskal-Wallis: χ^2^ = 13.79, d.f. = 2, P = 0.0010), posthoc tests found no significant difference between *Solanum* species. The fourth instar larvae distribution did not differ between the three *Solanum* species: out of the 11 individuals collected 72.7% were from *S. tuberosum* cv. *Désirée* and 27.3% from *S. polyadenium* (Kruskal-Wallis: χ^2^ = 4.14, d.f. = 2, P = 0.1263). The lower number of third and fourth instar larvae collected is probably the results of the *L. decemlineata* survey that ended only 16 days before the destructive sampling. During that sampling, all *L. decemlineata* eggs observed were removed and thus *L. decemlineata* population had only 16 days to build up again before the destructive sampling.

Aphids were sampled on 63 plants (21 plants by plant species). A total of 230 apterous aphids, all species confounded, were observed; 41.7% on *S. tuberosum* cv. *Désirée*, 41.7% on *S. polyadenium*, and 16.5% on *S. tarijense. Solanum tarijense* had significantly less apterous aphids than the two other species (Kruskal-Wallis: χ^2^ = 7.12, d.f. = 2, P = 0.0285). Most apterous aphids were too damaged (possibly because of either frost or alcohol) to be identified (68.3%).

**Table 2.**
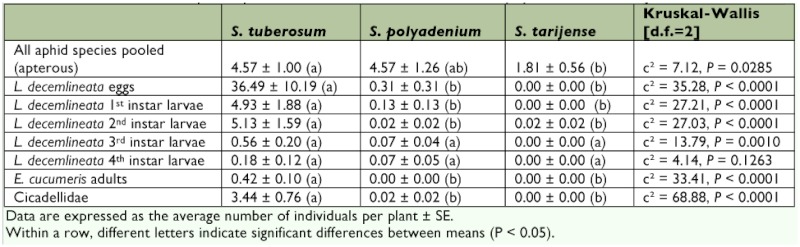
Mean abundance of potato pests on *Solanum tuberosum* cv. Désirée, *S. polyadenium*, and *S. tarijense*.

Natural enemy densities were very low in this study. A total of 74 spiders, 11 predaceous hemipteran nymphs, 3 predaceous hemipteran adults, and 1 coccinellid larva were collected from all three *Solanum* species.

## Discussion

Keeping aphids at low densities on potato plants is of primary importance as Mowry (2001) demonstrated that damages caused to tubers by PLRV are linearly correlated to *M. persicae* densities. The two experiments performed in this study showed that *S. polyadenium* and *S. tarijense* were generally more resistant than *S. tuberosum* cv. *Désirée* to the two most important aphid species present in Quebec, i.e. *M. persicae* and *M. euphorbiae*. As suggested by laboratory experiments (LeRoux et al. 2007, 2008), *S. stoloniferum* was also more resistant than *S. tuberosum* cv. *Désirée* to *M. persicae* in the field, but not to *M. euphorbiae*. On the other hand, *S. pinnatisectum* was found to be as susceptible to, or more susceptible than, *S. tuberosum* cv. *Désirée* to aphids. This contrast with Pelletier and Clark (2004) that demonstrated, in laboratory conditions, that the same accession of *S. pinnatisectum* was resistant to *M. euphorbiae*. This difference is possibly due to the impact of the environment on some resistance factors (Tingey and Gibson 1978; Obrycki and Tauber 1984; Gianfagna et al. 1992; Nihoul 1993) and highlights the importance of field experiments when evaluating pest resistance.

*Solanum tarijense* and *S. polyadenium* also had negative impacts on potato flea beetle and leafhopper populations. Apart from showing that *S. tarijense* is resistant to the flea beetle *E. cucumeris*, the results indicate that the observed resistance *of S. polyadenium* to *E. cucumeris*, and of *S. polyadenium* and *S. tarijense* to the leafhopper *E. fabae* (Sleesman 1940; Tingey and Gibson 1978; Flanders et al. 1992; Pelletier and Michaud 1995) also occurs in Quebec field condition.

More importantly, Colorado potato beetle, *L. decemlineata*, laid significantly less eggs on both *S. tarijense* and *S. polyadenium* than on *S. tuberosum* cv. *Desirée*. Again, the resistance of many *Solanum* species to *L. decemlineata* has previously been reported in other geographic areas, notably in New Brunswick, Canada (Pelletier et al. 1999; Pelletier and Dutheil 2006). Pelletier and Tai (2001) reported that the resistance mechanism of *S. polyadenium* PI 230463 (the same accessions) was mainly antibiosis as *L. decemlineata* laid more eggs on this species than on *S. tarijense* and other species. However, we observed significantly more *L. decemlineata* egg clutches on *S. tuberosum* cv. *Désirée* than on *S. polyadenium*, suggesting an antixenosis-based resistance in the wild *S. polyadenium*. Both experiments were done in field conditions but in different geographic areas, so the difference observed could be due to different growing or field conditions inducing differences in resistance factors.

For both *S. tarijense* and *S. polyadenium*, the difference in pest density was mainly conferred by the plant resistance characteristics since natural enemies' density was very low. This low density is not surprising as previous studies demonstrated that the density of most natural enemies in potato fields closely follows that of *M. persicae* and *M. euphorbiae* (Karley et al. 2003; Kabaluk et al. 2006). The relative capacity of pests and natural enemies to adapt and become able to exploit resources on resistant plant should be evaluated: should the pest adapt more rapidly, resistant plant would become an enemy-free space plant on which pests could thrive (Gassmann and Hare 2005). Particularly, *L. decemlineata* shows a strong adaptation capacity to locally abundant *Solanum* species (Hsiao 1978).

This study therefore supports the use of *S. tarijense* and *S. polyadenium* as candidate plants for hybridization with *S. tuberosum* in Quebec field conditions. However, further field experiments are still required to evaluate resistance in years of severe infestations of aphids or other pest species. Recently established in North America, *Aphis glycines* (Matsumura) has been reported to transmit potyvirus Y to potato plants (Davis et al. 2004, 2005). Future breeding programs should then evaluate resistance against this aphid species. Moreover, resistance factors to the most damaging bacteria, virus, and root-nod nematodes, as well as traits linked to vigour, have to be researched (Hawkes 1958). Finally, the impact of pests on yields should be studied as some resistant hybrids suffer more yield losses, even though pest densities are higher on susceptible varieties (De Medeiros et al. 2004).

In conclusion, these field experiments demonstrated the importance of *S. polyadenium* PI 230463 and & *tarijense* PI 414150 for breeding programs aiming at developing new pest resistant potato varieties. It also demonstrated the importance of field experiments in different geographic areas as resistance mechanisms may differ between field and laboratory conditions, and between geographic area.
